# The Janus Face of Stress on Reproduction: From Health to Disease

**DOI:** 10.1155/2015/458129

**Published:** 2015-04-07

**Authors:** Dóra Zelena

**Affiliations:** Hungarian Academy of Sciences, Institute of Experimental Medicine, Szigony 43, Budapest 1083, Hungary

## Abstract

Parenthood is a fundamental feature of all known life. However, infertility has been recognized as a public health issue worldwide. But even when the offspring are conceived, in utero problems can lead to immediate (abortion), early (birth), and late (adulthood) consequences. One of the most studied factors is stress. However, stress response is, per se, of adaptive nature allowing the organism to cope with challenges. Stressors lead to deterioration if one is faced with too long lasting, too many, and seemingly unsolvable situations. In stress adaptation the hypothalamus-pituitary-adrenocortical axis and the resulting glucocorticoid elevation are one of the most important mechanisms. At cellular level stress can be defined as an unbalance between production of free radicals and antioxidant defenses. Oxidative stress is widely accepted as an important pathogenic mechanism in different diseases including infertility. On the other hand, the goal of free radical production is to protect the cells from infectious entities. This review aims to summarize the negative and positive influence of stress on reproduction as a process leading to healthy progeny. Special emphasis was given to the balance at the level of the organism and cells.

## 1. Introduction

Parenthood is one of the most universally desired goals of adulthood in all species [1]. However, among humans not all couples who desire a child will achieve one spontaneously. Although global infertility prevalence rates are difficult to determine due to the presence of both male and female factors [2], infertility has been recognized as a public health issue worldwide by the World Health Organization [3]. One in every four couples in developing countries had been found to be affected by infertility. Around 50% of these cases are due to males, while the other half is due to female factors [4, 5]. Thus, the burden remains high. For better understanding of the biological processes and for developing new treatment strategies animal models are indispensable. Moreover, there is an economic interest due to livestock and plant infertility.

*Reproduction*. Reproduction is a fundamental feature of all known life; each individual organism exists as the result of reproduction and its main goal is to reproduce its gens [6]. In* asexual* reproduction an individual can reproduce without involvement of another individual of that species [7].* Sexual* reproduction typically requires the involvement of two individuals or gametes. There is a wide range of reproductive strategies [8]. Some animals such as the human do not reach sexual maturity for many years after birth and even then produce few offspring (*k-strategy*). For example, elephants have one baby about three years apart, and the whole group looks after the youngsters. Because they ensure the survival of a good percentage of their young, elephants do not need to produce many offspring to hold their numbers close to constant. Other species reproduce quickly; however, under normal circumstances, most offspring do not survive to adulthood (*r-strategy*). Frogs are a good example as they lay many eggs and leave them in the water to hatch into tadpoles. Some of the eggs and many of the little tadpoles as well as developed frogs get eaten; thus, if one frog from a hundred eggs lives to be a parent, his/her survival is really outstanding. However frogs survive because of the many eggs.

In a wider view reproduction is a process leading to healthy progeny. This process can be influenced by internal and external factors. First of all several factors have an impact on fertility and mating ability of the individuals. But even when the offspring are conceived, in utero problems can lead to immediate (abortion), early (detectable at birth, e.g., preterm birth, low birth weight, and malformations), and late consequences (detectable later in life and might be only changes in vulnerability) [9]. The “developmental origins of health and disease” concept says that the risk of developing some chronic diseases in adulthood is influenced by environmental factors acting during the periconceptual, fetal, and infant phases of early life [10].

Nowadays human beings are challenged by changing in mother-child relationship and social models of the extended family [11]. The accelerated pace of life and unpredictable environmental changes are added to the above factors. All of these factors are, actually, chronic stressors, which we have to deal with and they influence the reproduction.

*Stress*

*In the Body*. Numerous health experts say that the number one killer on the planet is stress. On the other hand the father of stress concept, Hans Selye, considered stress the “spice of life” [12]. As such, he felt it was neither advantageous nor even possible to eliminate it from life. Instead, the challenge is to contain any stress and channel it into feelings of mastery.

**Figure 1:**
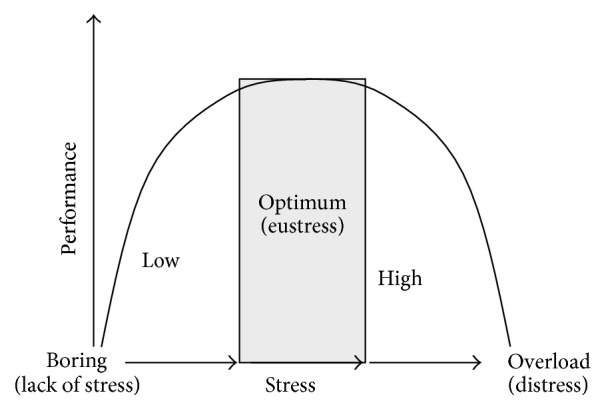
Stress in the body: an optimal level of stress (called eustress) is required for the best performance. Not only too much, but also too little stimuli are harmful.

We have to take into consideration a number of factors contributing to the qualitative nature of the stress response such as the intensity (high or low) and duration (acute or chronic) of stressors, the individual's ability to initiate an adaptive response, and the phase of the life when the stressor event occurs [13]. When we can cope with the challenges, then we can become stronger (Figure 1). Thus, stress response is, per se, of adaptive nature as it allows the organism to cope with stressful challenges, no matter of what nature, and to maintain or restore body homeostasis. This is considered to be* eustress*, which has also been positively correlated with life satisfaction and well-being. On the contrary, when a person is unable to adapt to the stressors, they will induce maladaptive behaviors. We can call this state* distress*. We have to be also aware that the complete lack of stimuli is also harmful. Thus, although it is important to minimize your distress, yet at the same time you have to maximize your eustress by optimizing the amount of stimuli.

**Figure 2:**
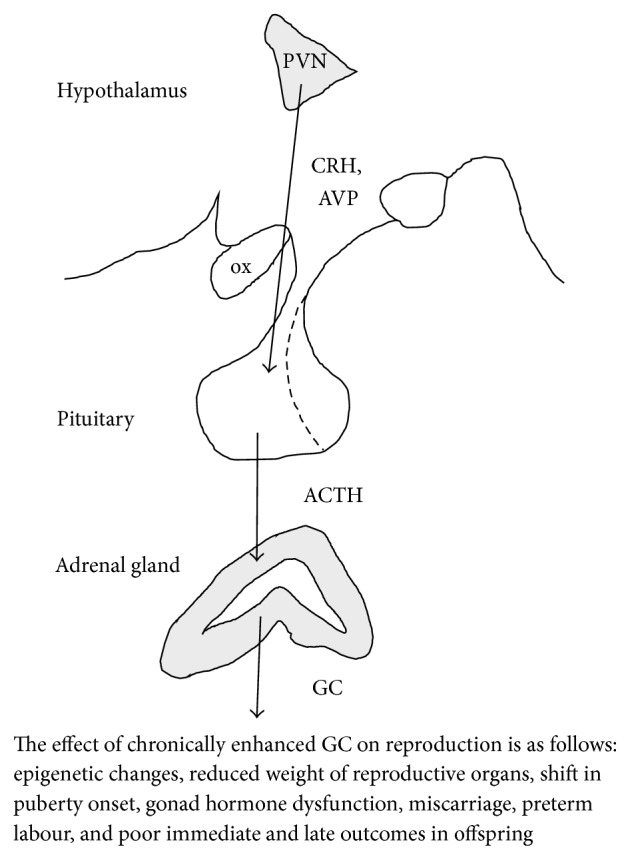
The stress axis: the activation of the hypothalamo-pituitary-adrenocortical axis is fundamental for adaptation. PVN: nucleus paraventricularis hypothalami, ox: optic chiasm, CRH: corticotropin-releasing hormone, AVP: arginine vasopressin, ACTH: adrenocorticotropin, and GC: glucocorticoids, in rodents corticosterone.

During stress several systems are activated in the body; however, according to Selye, the hypothalamus-pituitary-adrenocortical (HPA) axis, as well as the resulting glucocorticoid (GC) elevation, is the most important one (Figure 2). As a final common pathway to different kind of stimuli corticotropin-releasing hormone (CRH) and arginine vasopressin (AVP) are released from the parvocellular cells of the nucleus paraventricularis hypothalami (PVN) to the long portal vessels of the median eminence [14]. They reach the anterior lobe of the pituitary through these vessels and stimulate the synthesis and release of adrenocorticotropin (ACTH) from the adenohypophysis. ACTH reaches the adrenal gland through the general circulation and releases GCs (mainly cortisol in human and corticosterone in rodents) from the zona fasciculata of the adrenal cortex. The release of GCs is an adaptive response with an ultimate goal to maintain homeostasis. HPA axis activation favors energy mobilization, cardiac output, and sharpened cognition over growth, cellular immunity, and reproduction. Thus, at high circulating levels of GCs survival occurs at the expense of reproduction. Therefore we can conclude that the division of resources in the trade-off between survival and reproduction is likely mediated, in part, through regulation of the stress response by the HPA axis [15].

**Figure 3:**
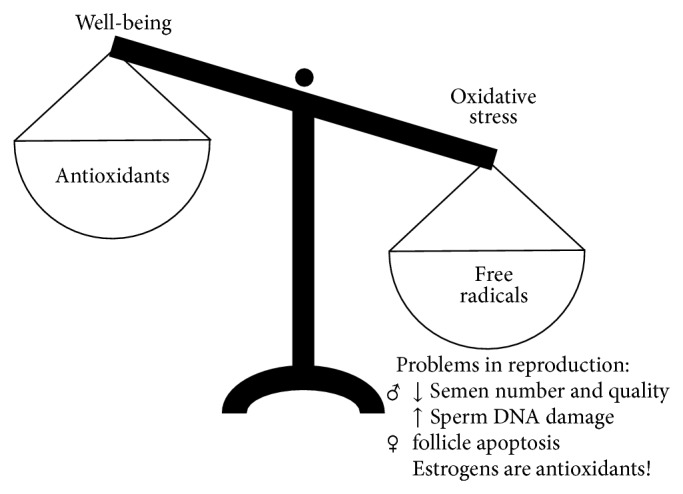
Stress at cellular level: an unbalance between production of free radicals (e.g., reactive oxygen species (ROS)) and antioxidant defenses inducing oxidative stress in the cells. Although oxidative stress is widely accepted as an important pathogenic mechanism in different diseases, yet ROS are physiological mediators of transcription control and in many cells (e.g., leukocytes) ROS are protection factors against infectious stimuli. The complete elimination of free radicals would thus disrupt, rather than extend, the normal functioning of the body.

*At Cellular Level*. Beyond the general beliefs stress can be defined also at cellular level as an oxidative stimulus. Oxidative stress (OS) is an unbalance between production of free radicals, molecules characterized by high reactivity (like reactive oxygen species (ROS) such as superoxide anion, hydrogen peroxide, and the hydroxyl radical) and antioxidant (such as glutathione, vitamins, catalase, superoxide dismutase, and various peroxidases) defenses in biological systems (Figure 3) [16]. Enhancement in ROS and their metabolites can attack the cell membrane, and they can result in modification of the DNA, lipids, and proteins (such as tyrosine nitration and S-glutathionylation), altering enzymatic systems. The imbalance of ROS can eventually lead to epigenetic differences, changes in cellular pathways and transcription factors, produce irreparable alterations, and cause cell death [4]. OS is widely accepted as an important pathogenic mechanism in different diseases like rheumatoid arthritis, myocardial infarction, diabetes mellitus, and so forth [17]. According to the free radical theory (also known as the OS theory) of aging the progressive decline in physiological functions with age is also a result of the accumulation of ROS-induced damage [18]. Among others OS has been identified as one of the many mediators of infertility [19].

On the other hand, in leukocytes and many other cells (endothelial cells, mesangial cells, fibroblasts, thyrocytes, oocytes, Leydig cells, adipocytes, etc.) ROS generation has been assessed to have a positive physiological role protecting the cells from pathological stimuli [16].

## 2. Janus Face of Stress

### 2.1. Negative Effects

Individuals have limited resources that they can either devote to reproduction or devote to somatic maintenance (e.g., protection against ROS) [20]. Therefore it is not surprising that the general view is that stress, which requires efforts to maintain homeostasis, negatively influences reproduction.

#### 2.1.1. Fertility

Stress-induced trade-offs to reproductive output are evident across species. Animals (mammals, birds, and reptiles), but even plants, all respond to physical stress by decreasing male and female reproductive function [15, 21]. From economical point of view, summer heat stress is a main factor related to low fertility in high-producing dairy herds living in warm areas worldwide [22]. Costs of reproductive effort are also evident: adult female rodents and primates have higher GC levels during lactation [23], which may lead to the development of stress-related disorders like anxiety and depression [24]. Negative consequences of stress are also present in human and are not only an acute, short lasting effect on fertility or mating abilities; but psychological or physical stress experienced by adolescents can alter the onset of puberty, shifting the clock of fertility [25] (Figure 2).

Considering the mechanism of stress-induced infertility, expression of glucocorticoid receptors (GRs) has been described in multiple cell types of the testis and expression is conserved across species. The male gonads are direct targets of GC action controlling testosterone biosynthesis in Leydig cells [26]. Additionally, exogenous and stress-induced GCs cause Leydig cell death [27, 28], thereby providing contributing mechanisms to stress-derived androgen dysfunction in males. The role of GCs in the female reproductive tract is realized in the hypothalamus, where it is well established that the stress-activated HPA axis suppresses hypothalamic-pituitary-gonadal (HPG) function through influencing the gonadotropin releasing hormone secretion. In addition to direct HPG axis regulation, GCs may regulate novel mediators of the axis like gonadotropin-inhibitory hormone and kisspeptin. Moreover, GRs have also been demonstrated in the ovary, where GCs directly regulate steroid biosynthesis.

Another possible contribution of stress to fertility is at cellular level. Human-made chemicals (among other air pollutants) as well as unhealthy lifestyle behaviours, mainly obesity, tobacco smoking, alcohol consumption, and medical drug abuse, involve the generation of ROS and cellular oxidative damage. More data are available on male reproduction as both spermatogenesis and Leydig cell steroidogenesis are very vulnerable to OS [17]. The impairments in male infertility have resulted via mechanisms involving the induction of peroxidative damage in the sperm plasma membrane, DNA damage, and apoptosis [19, 29] leading to metabolic and functional disorders of male germ cells and may be a primary cause of some types of infertility [30]. Motor vehicle exhaust contains variety of toxic components and contributes to a large proportion of the air pollution [31]. Its inhalation can cause harmful effects on male reproductive functions by altering organ weights, reducing the spermatozoa qualities, and inducing OS. On the other hand, suboptimal maternal nutrition (both low-protein diet and maternal obesity) is accompanied by enhanced OS in offspring leading to altered sperm function too [32, 33]. Not only in rodents, but also in humans, paternal obesity is associated with increased OS in sperm, reduction in semen quality, and decreased fertility. Beside direct oxidative damage the OS-induced decrease in fetal lutein hormone concentration has negative consequences on gonadal development leading to decreased testosterone serum levels and sperm concentration [33].

Although females are not that well studied, yet there is strong evidence that ROS are involved in ovarian toxicity as well [4, 34]. More specifically, several chemical and physical agents induced ROS production, which initiates apoptosis in antral follicles and also in primordial and primary follicle [35]. We have to add, however, that ovarian steroid might have protective role by upregulating the expression of antioxidants [36]. Indeed, many preclinical reports indicated an enhancement in OS after ovariectomy [37] and in postmenopausal women estradiol therapy might decrease the ROS production [38].

Another side of the coin is that free radicals and ROS are produced in direct proportion to metabolic rate as an inevitable consequence of the molecular functioning of mitochondria and the electron transport chain. (This notion was subsequently encapsulated in the “rate of living” theory [39], the idea that living fast is inevitably linked to dying young.) Higher rates of metabolism that accompany reproduction lead to greater free radical production and hence act as a potential mediator of the trade-off between reproduction and survival [20]. This cost of reproduction was confirmed in many different species. For example, an increase in* Drosophila melanogaster* female reproduction increased the susceptibility to OS [40].

#### 2.1.2. In Utero Stress

Spontaneous miscarriage is the most common adverse pregnancy outcome in humans and occurs in 15–20% of all recognized pregnancies [41] (Figure 2). The causes for a spontaneous miscarriage are diverse and comprise genetic, endocrinologic, anatomic, immunologic, or microbiologic aspects. Among others, environmental factors such as psychosocial stress have been identified to account for infertility and unexplained reproductive failure in humans and other mammalian species such as baboons, elks, and rodents. Stress has been shown to challenge pregnancy maintenance by initiating an inflammatory response at the fetomaternal interface.

High perceived stress during pregnancy is a risk factor for preterm labor and poor outcomes in offspring [42]. Moreover, a higher frequency of growth-restricted fetuses is a well-documented side effect of GC treatment during pregnancy [43]. Although some form of stress per se might not be harmful (e.g., work-related occupational stress), the presence of other risk factors may result in a synergistic effect which strengthened the odds of an adverse outcome [44]. It was also established that, for example, disasters as perinatal stressors themselves are not that influential on child development, but the mental health of the mother (disturbed by the disaster) may strongly influence the later development [45].

The consequences of intrauterine stress extend beyond the immediate perinatal period. Epidemiologic studies suggest that elevations in GC exposure during fetal life may result in fetal programming of cardiovascular, metabolic, and neuroendocrine disorders in adult life. In the placenta, conversion of maternal GCs into their inactive metabolites provides a protective barrier. However, following GC excess, this may be ineffective in protecting the fetus from overexposure. In the rat, maternal exposure to dexamethasone (a synthetic GC) during gestation results not only in reduced birth weight, but also in hypertension of the adult offspring [46, 47]. A nonhuman primate model of intrauterine dexamethasone treatment revealed a dose-dependent reduction in postnatal growth together with impaired glucose tolerance and hyperinsulinemia in offspring [48]. Similarly, antenatal GCs treatment in human offspring also induced insulin resistance [49]. Furthermore, fetal exposure to synthetic GCs is associated with persistent neurological consequences in humans, including cortical thinning [50]. Excessive endogenous GC levels have similar consequences (reduced birth weight and programming effects, e.g., emotional problems) [51, 52]. One of the underlying mechanisms could be a change in hippocampal GR expression, an important component of the negative feedback loop of the HPA axis [53].

As late, transgenerational consequences, offspring of female rats subjected to restraint or environment stress (e.g., nutrient restriction) during pregnancy experienced fewer and longer pregnancies, with less viable young, as adults [54–56]. Male rats from stressed or GC treated dams also demonstrated reduced sexual behavior and fertility [57, 58]. An indirect connection between in utero stress and reproduction was that stress, ACTH, or GC treatment of the mice mother during pregnancy induced a subsequent delay in the onset of puberty [59]. Persistent changes in gene expression that remain after the inciting event are thought to be mediated by epigenetic mechanisms [60]. Intrauterine growth restriction in the rat caused changes in epigenetic profile of GR in the hippocampus [61]. Moreover, fetal GC exposure caused significant variations in genome-wide promoter methylation, ultimately leading to profound changes to the epigenetic landscape.

Additionally, not only perinatal stress of the pups [14] but also maternal stress during lactation may alter the development of the offspring [62].

### 2.2. Positive Effects

#### 2.2.1. Fertility

GCs mediate the release of sex steroids thereby regulating the timing of puberty onset. However, an optimum level is needed, as both in the case of hypocortisolism (Addison's disease) and in the case of hypercorticism (Cushing's syndrome) the onset of puberty is altered.

It is known that preconceptional effects can profoundly alter later pregnancy and the fetus [9]. Despite this fact, in humans, the pregnancy rate is not influenced by injection drug (such as heroin) use [63], known to stimulate the HPA axis [64]. We must mention, however, that low uptake of reliable contraception can confound these results. Nevertheless, appropriate usage of morphine can be even beneficial in enhancing the pregnancy rate, based on a study using embryo transfer in rats [65].

Moreover, physical exercise, which stimulates the HPA axis as well [66], may prevent reproductive complications associated with maternal obesity. The reduction in obesity lowers the risk of infertility and miscarriage, and it may also reduce the probability of obesity-related complications during later pregnancy [67].

A possible mechanism of the enhanced pregnancy rate can be the apoptosis inhibiting effect of GCs on luteal cells, as the maintained luteal function contributes to the survival of the fetus [68].

As a further positive effect at homeostatic levels GCs regulate many key aspects of early pregnancy, including the effects of the maternal immune system, embryo attachment, and invasion, along with the growth and development of the fetus [15, 69]. Despite the previously mentioned negative consequences, the pregnancy-promoting potential of GCs was also confirmed [70]. It is well established that uterine receptivity and embryo implantation are determined by uterine natural killer (NK) and other cells regulated by the immune system [71]. Daily GC administration suppressed the levels of uterine NK cells in a patient with a history of recurrent miscarriages [72]. These immune modulatory effects of GCs provide a link between stress and reproductive function through improvement of the intrauterine environment. Although some further human cases also suggest positive pregnancy rates and outcomes after preconceptional GC treatment [73, 74], yet the association is not that unequivocal [75]. Nevertheless, there are GRs in the uterus and GCs regulate estrogen effect through an interaction with estrogen receptors. Moreover, in relation with reproduction GCs primarily induce an antiapoptotic effect in cells of the mammary gland [15].

With respect to the cellular level, some carefully controlled laboratory work has repeatedly concluded that OS is unchanged or is lower in those individuals that reproduce compared to those that do not despite the reproduction-survival trade-off (see earlier) [76]. Females have extremely high energetic demands during reproduction, particularly through lactation. However, there was no evidence of increased OS in mice. Instead, in the liver, markers of oxidative damage (malondialdehyde, protein thiols, and the proportion of glutathione in the oxidized form) indicated lower OS in reproducing females when compared with nonreproductive controls. Even during peak lactation, none of the markers of oxidative damage indicated higher OS than among nonreproductive females. Another example is the breeding canaries, which showed decrease in plasma oxidative damage (reactive oxygen metabolites and protein carbonyls) compared both with nonbreeding canaries and premanipulation values irrespective of sex and brood size [77].

The reason might be that, for several transcription factors, ROS are physiological mediators of transcription control. The well-known examples of redox-sensitive transcription factors are nuclear factor-*κ*B (NF-*κ*B) and activator protein-1 (AP-1). Studies have also demonstrated that low and controlled concentrations of ROS play an important role in normal sperm physiological processes such as capacitation, hyperactivation, acrosome reactions, and signaling processes to ensure appropriate fertilization [19]. The complete elimination of free radicals would thus disrupt, rather than extend, the normal functioning of the body.

Another explanation is that reproduction activates behavioural or physiological mechanisms that protect against OS in order to improve immediate reproductive success and, possibly, not to compromise future reproduction. This may be especially important in species with multiple reproductive cycles over the course of a lifetime.

Moreover, oxidative damage is not necessarily an evolutionary mistake but may be beneficial for evolution [78].

#### 2.2.2. In Utero

GCs are essential in mammals toprepare for life after birth. Their blood levels rise dramatically shortly before birth. GCs trigger parturition not only in animals, but also in humans [79]. However, for labour simultaneously high estrogen levels are also required, but GCs may inhibit the synthesis of the precursors both in fetus and mothers, thereby reducing the placental estrogen synthesis. Thus, the exogenously given GC may not be beneficial; however, the estrogen inhibiting effect of endogenous GC might be counteracted by its degradative enzymes (11-beta-hydroxysteroid dehydrogenase) in the placenta.

If there is any sign of preterm birth, it has become common practice to treat either antenatally the mother or postnatally the infant with GCs to accelerate tissue development, particularly of the lung [80]. This is a rather beneficial effect; however, in the long run it can increase the risk of adverse outcome in later life or even shorter lifespan (see earlier). The perinatal action of the GCs depends on the context and the timing as well as the type of administered steroid. The optimal amount of GCs is very important as either insufficient or excessive GC exposure before birth may alter the normal GC-regulated trajectory of maturation with potential life-long consequences [81].

Controversially, enriched environmental model, where the animals show all endocrine signs of a chronic stress state [82], was beneficial in many preclinical disease models [83, 84]. However, prenatal enriched environment (through the entire pregnancy) per se induced anxiety and depressive-like behaviors with poor attentional performance in male adult offspring [85]. On the other hand it prevented the harmful effect of adult acute stress. Moreover, juvenile enrichment could transgenerationally rescue a genetic defect in long-term potentiation and memory in a knockout mouse strain [86]. Thus, prenatal enriched environment may be also beneficial leading to resilience to adulthood.

Another model leading to chronic stress is the voluntary wheel running [66, 87]. The beneficial effect of physical activity in many disease states is well-documented. In rodents, wheel running during pregnancy transiently enhanced memory and hippocampal neurogenesis in the offspring until preadolescence [88, 89]. Exercise during pregnancy provided long lasting protection from neurodegeneration and improved brain plasticity in the otherwise unstimulated progeny [90]. Moreover, maternal voluntary wheel running significantly offset morphological impairments due to prenatal stress (maternal restraint) in the offspring [91]. In humans, maternal exercise during pregnancy positively influenced fetal health and offspring's cognitive performance until childhood [92]. Women who are the most physically active had the lowest prevalence of gestational diabetes with decreased incidence of obesity and type 2 diabetes in both mother and offspring [67]. Exercise during pregnancy and lactation prevented maternal obesity-induced elevation in corticosterone in rat offspring as well [93]. Thus, in addition to maintaining physical fitness in the pregnant women, exercise may be beneficial in preventing or treating maternal-fetal diseases.

Although in utero stress mostly results in low birth weight, yet some authors reported even increased birth weight after intrauterine protein restriction [94]. We have to add, however, that even in this case the pups had proportionally smaller brains and at weaning rats exposed to low-protein diets in utero had significantly higher systolic blood pressure relative to control animals.

The long-term consequences of in utero stimuli might be dependent on the later environment. For example, the offspring of protein restricted mothers lived longer than absolute controls if they were to grow up on protein restricted diet; however, a normal diet shortened their life [56]. This effect might be induced by predictive adaptive response (a kind of preconditioning) [10]. It means that the organism adapts to a certain environment in utero and in case after birth the eventually experienced environment matches the predicted environment, the organism's phenotype proves to be adapted and life span is extended.

Positive effect of stress was detected also in the case when repeated maternal stress was applied during the lactation period [95]. Male offspring of these mothers favoured goal-directed behaviours and showed enhanced behavioural flexibility in their adulthood. Additionally, when the mothers' drinking water was supplemented with a low dose corticosterone (0.2 mg/mL) during the lactation (reflecting a form of mild environmental stimulation), the offspring develop the ability to cope better with different situations during life [13]. The progeny of these mothers, once adults, showed improved learning capabilities, reduced fearfulness in anxiogenic situations and resistance to ischemic neuronal damage, and adapted better to colonic inflammatory stress. This protective effect is linked to hyporeactive HPA axis due to epigenetically transmitted enhanced negative feedback in the hippocampus.

## 3. Intervention Options

Taking into consideration thegrowing infertility public health issue worldwide it is important to prevent the negative consequences of stress on reproduction.

### 3.1. Body Stress

Because stress is considered to be number one killer, several methods (many belonging to alternative medicine) have been developed for stress relief. It is clear that the mental status of the parents can profoundly alter the fertility [96, 97] and stress has a great impact on the psyche [24]. Therefore mental well-being is an important factor [98], which can be reached by different relaxations techniques (mental or physical), nutritional interventions, and pharmacological treatments. Psychological interventions were found to improve some patients' chances of becoming pregnant [99] and relaxation may prevent preterm labour [100]. Massage therapy may be also relaxing [101]. Physical activity is a good example of eustress [67]. The beneficial effect of exercise [102] may be due—at least partly—to its trophic effect through, for example, an enhanced brain derived growth factor level in the hippocampus [103]. Pharmacological intervention is focused mainly on the treatment of stress-related disorders, thereby preventing secondary diseases. For example, pain killers [104], anxiolytics [105], and antidepressant are all good for stress relief, thereby enhancing the spirit for reproduction, but their potential for enhancing, for example, the pregnancy rate is not known, yet.

### 3.2. Oxidative Stress

Already plants have evolved a plethora of mechanisms to circumvent the potential damaging effects of OS. These mechanisms include different levels of organization, from root-shoot signalling at the whole-plant level to specific biochemical responses at the subcellular level, such as reductions in photosynthesis and the consequent activation of photo- and antioxidant mechanisms in chloroplasts [106].

In mammals different antioxidant systems help to protect the body against OS. For example, the MRE11 complex is an important source of stress relief, being a key component of DNA repair [107]. The most studied antioxidants are ascorbic acid (Vitamin C), tocopherols (Vitamin E), and carotenoids (Vitamin A), but enzymes, as well as Vitamin B complex, glutathione, pantothenic acid, coenzyme Q10, and carnitine, and micronutrients such as zinc, selenium, and copper seem to be also important [29, 108, 109]. However, only low quality evidence suggests that antioxidant supplementation in subfertile males may improve clinical pregnancy rates [5] and in pregnant women there were no changes in smoking induced OS in relation to vitamin supplement intake [110].

Because of high incidence obesity is one of the most prevalent sources of OS [32]. Weight loss inducing lifestyle changes such as dietary and exercise interventions has beneficial effects on metabolism both in rodents and in humans [102]. In obese men weight loss can improve semen parameters and therefore fertility.

## 4. Conclusion

As many other processes, stress has also two faces; it is at the same time the good and the bad guy. We have to be aware that the most important thing is the balance, not too much, but also not too little (Figure 1).

A stress situation in itself does not affect health harmfully when it is accompanied by the feeling that one is able to overcome difficulties successfully, which is actually the basis of physical and mental development [11]. Challenges and new situations lead to deterioration if one does not know the solutions, if one is faced with too long lasting, too many, and seemingly unsolvable situations, and if society seems unpredictable, chaotic, and uncontrollable. The distress is the hotbed of reproductive disabilities as well. However, eustress, a certain level of HPA axis activation, is important for normal fertility and pregnancy as well as intrauterine and later development. An optimal level of stress is required for resilience to bigger, harmful challenges.

At cellular level, when the natural balance between ROS and antioxidants is disturbed, the first restorative measure to be taken should be changes in lifestyle, such as cessation of smoking, limiting substance use, and maintaining a healthy and balanced diet, which all may increase the reproductional capacity. Antioxidant supplementation may be taken to improve the patient's health outcomes including fertility, pregnancy rate, and normal pregnancy [19]. We have to be aware that males are more vulnerable to OS than females because of the protective role of estrogens. On the other hand, it appears that threshold levels for the benefit or harm of OS exist. Therefore care must be given to acknowledge potential undesirable effects of excessive vitamin supplementation [111].

## Abbreviations

ACTH: Adrenocorticotropin
AP-1: Activator protein-1
AVP: vasopressin
CRH: Corticotropin-releasing hormone
GC: Glucocorticoid
GR: Glucocorticoid receptors
HPA: Hypothalamic-pituitary-adrenocortical axis
HPG: Hypothalamic-pituitary-gonadal
NF-*κ*B: Nuclear factor-*κ*B
NK: Natural killer
OS: Oxidative stress
PVN: Nucleus paraventricularis hypothalamic
ROS: Reactive oxygen species.
